# Optimal timing of blastocyst vitrification after trophectoderm biopsy for preimplantation genetic screening

**DOI:** 10.1371/journal.pone.0185747

**Published:** 2017-10-05

**Authors:** Hsiu-Hui Chen, Chun-Chia Huang, En-Hui Cheng, Tsung-Hsien Lee, Lee-Feng Chien, Maw-Sheng Lee

**Affiliations:** 1 Division of Infertility, Lee Women’s Hospital, Taichung, Taiwan; 2 Department of Life Sciences, College of Life Sciences, National Chung Hsing University, Taichung, Taiwan; 3 Institute of Medicine, Chung Shan Medical University, Taichung, Taiwan; 4 Department of Obstetrics and Gynecology, Chung Shan Medical University Hospital, Taichung, Taiwan; 5 Department of Obstetrics and Gynecology, College of Medicine, National Taiwan University, Taipei, Taiwan; University of Otago, NEW ZEALAND

## Abstract

Is the timing of vitrification after trophectoderm (TE) biopsy associated with successful implantation and pregnancy after the embryo transfer of blastocysts subjected to preimplantation genetic screening (PGS)? In this retrospective cohort study, 1329 blastocysts from 223 patients were subjected to TE biopsy for performing array comparative genomic hybridization (CGH) tests. The PGS and frozen blastocyst transfer (FET) cycles were performed from December 2012 to May 2015. Only the good quality and expanded blastocysts on day 5 or 6 were selected for biopsy. After TE biopsy, the re-expansion grades relative to the original blastocoel were (1) collapsed blastocysts (CB), (2) 3/4 re-expansion but not full expansion (RE), and (3) full re-expansion or hatching (FE). All biopsied blastocysts were subjected to vitrification within 0.5–6 h after biopsy; the time intervals between TE biopsy and vitrification and the expansion grades at the time of vitrification were recorded. By combining two factors, namely the expansion grades and culture intervals between biopsy and vitrification, the patients were further divided into four groups, namely CB with a < 3 h culture interval (n = 34 cycles, Group I), RE and FE blastocysts with a < 3 h culture interval (n = 10 cycles, Group II); CB blastocysts with a ≥ 3 h culture interval (n = 6 cycles, Group III); and RE or FE blastocysts with a ≥ 3 h culture interval (n = 173 cycles, Group IV). The implantation (63.7%, 179/281) and clinical pregnancy (74.0%, 128/173) rates in Group IV were significantly higher than those in Group I (45.3%, 24/53; 50.0%, 17/34; P = 0.012 and 0.005, respectively). According to our findings, optimal vitrification timing > 3 hours to enable blastocysts to reach RE or FE provides improved implantation and pregnancy rates after FET.

**Trial registration:** ClinicalTrials.gov NCT03065114

## Introduction

The blastocyst stage is the optimal stage for performing biopsies for preimplantation genetic screening (PGS) [1]. The high efficiency of PGS at the blastocyst stage has being reported as a key factor determining the growing clinical application of this strategy worldwide [2–7]. For blastocyst biopsy, laser-assisted drilling is used to create a zona opening on day 3 or day 5 of development. The method of zona opening on day 3 allows some of the TE cells to herniate during blastocyst formation and expansion, which facilitates the biopsy process. However, this method may result in herniation of inner cell mass (ICM) cells instead of TE. In contrast, the method of zona opening on day 5 is performed at the expanded blastocyst stage with a hole drilled away from the ICM to ensure that only TE cells are biopsied. The disadvantage of this method is an obvious blastocyst collapse after TE biopsy. The collapsed blastocyst requires some time to seal the hole where the cells have been removed and reform the blastocoel [8–10].

Biopsied blastocysts are routinely stored in liquid nitrogen for subsequent transfers. Blastocyst vitrification in the routine management of in vitro fertilization (IVF) cycles enables performing trophectoderm (TE) biopsy with high efficiency and minimal risks [3, 11, 12]. In past years, the clinical outcomes of PGS were focused on the biopsy stage to reduce embryo damage or technical risk (mosaic or allele dropout) [13]. However, the most appropriate time prior to vitrification following TE biopsy for PGS-FET remained unclear.

The TE epithelium forms on the outside of blastocysts to act as a barrier and regulate the exchange and accumulation of small molecules and fluid during blastocoel formation [14]. TE biopsy typically alters the integrity of tight junctions (TJs) and causes morphological changes (blastocyst collapse and cells loss) because of decreased pressure. The TJs between TE cells are an essential component of the differentiated epithelial cells required for polarized transport, intercellular integrity and signaling [15]. By contrast, the integrity and formation of the blastocyst cavity (blastocoel) is maintained by the sodium pump (Na+/K+-ATPase), which gradually increases fluid accumulation and pressure on both TE and zona pellucida (ZP) [16]. The collapsed blastocoel gradually re-expands regaining fluid accumulation and pressure on both the TE and ZP. Hatching is facilitated by robust expansion of the TE and blastocoel during preimplantation development. The embryo is released from the ZP which increases the efficiency of interaction with the uterine endometrium during implantation [17]. If the vitrification of biopsied blastocysts is performed immediately after TE biopsy, it is impossible to know the impact of embryo recovery status. The degree of re-expansion is one indicator of recovery status for biopsied blastocysts, which may relate to the PGS-FET efficiency. However, according to our review of relevant literatures, the relationship between the re-expansion grade after TE biopsy and clinical outcomes of PGS—FET has not been previously discussed.

This study determined whether (1) re-expansion grades of biopsied blastocysts at the time of vitrification, (2) the culture interval between TE biopsy and vitrification, and (3) a combination of both parameters is associated with implantation and pregnancy outcomes in PGS—FET cycles. The findings are useful for further enhancing the clinical outcomes obtained from vitrified euploid embryos.

## Materials and methods

### Patient selection

**Fig 1:**
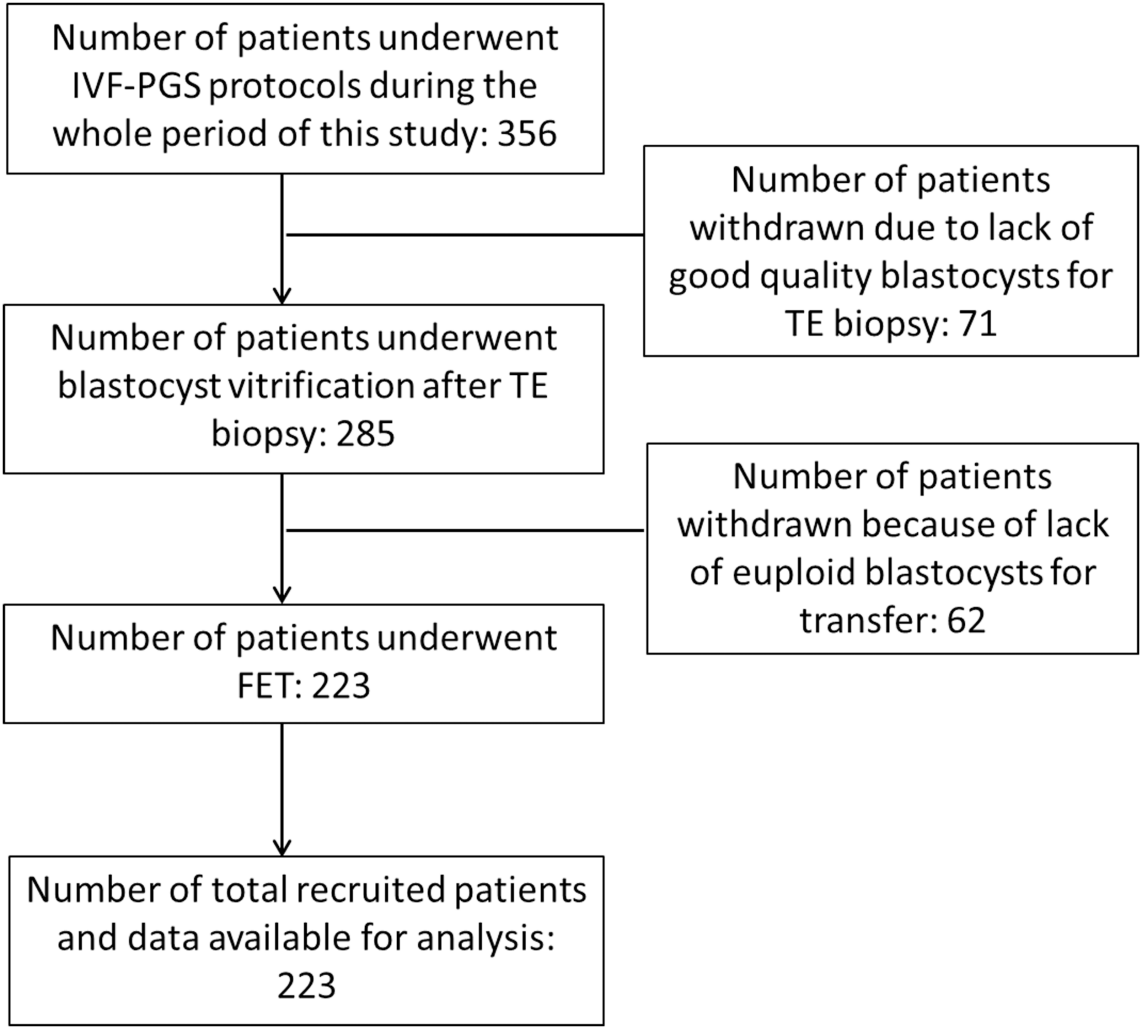
Retrospective cohort study design and study flow chart.

This is a retrospective, single-center clinical trial to evaluate the effect of vitrification timing after TE biopsy. Fig 1 is a flow chart demonstrating the study design. The study cohort comprised a total of 223 women referred to Lee Women’s Hospital, Taiwan and treated with PGS and FET from December 2012 to May 2015. All patients and their husbands signed standard IVF consent forms and underwent the same stimulation and FET protocols. All aneuploidy screenings were authorized by patients after consultation. All treatment history and clinical outcomes of patients were recorded in the database system of Lee Women’s Hospital before analysis. The staff member performing the database recording and assessments was not involved in implementing any aspect of the intervention and knew the participants only by their study identifier number. The exclusion criteria were as follows: (1) an age > 39 years, (2) repeated implantation failure, (3) recurrent miscarriage, and (4) sperm donation. The diagnosis of exclusion criteria was according to reports of attending physicians at Lee Women’s Hospital. In this study, vitrification-warming of the biopsied blastocysts was performed by the same embryologist to reduce the impact of technical variation on the clinical results. After data collection, the PGS cycles for analysis were divided into several subgroups according to two criteria: (1) re-expansion grade of blastocyst and (2) duration time of blastocyst re-expansion after TE biopsy. The retrospective data analysis was approved by the Institutional Review Board of Chung Shan Medical University, Taichung, Taiwan (CS-14124).

### In vitro fertilization protocol

Controlled ovarian stimulation, oocyte collection, and denudation were performed as previously described [18]. All patients were administered leuprolide acetate (Lupron, Takeda Chemical Industries, Ltd., Osaka, Japan), started during the midluteal phase for downregulation. All patients subsequently received recombinant follicle stimulating hormone (Gonal-F; Serono, Bari, Italy) from cycle day 3 for ovarian stimulation until the dominant follicle reached a diameter of > 18 mm, followed by injection of 250μg human chorionic gonadotropin (Ovidriell, Serono) 36 h before oocyte retrieval.

### Embryo culture

The retrieved oocytes were cultured in Quinn’s Advantage Fertilization Medium (Sage BioPharma, Inc., Trumbull, CT, USA) with 15% serum protein substitute (SPS, Sage BioPharma, Inc) in a triple gas phase of 5% CO_2_, 5% O_2_, and 90% N_2_. Following conventional insemination or intracytoplasmic sperm injection (ICSI), all embryos were further cultured in microdrops of a cleavage medium (Sage BioPharma, Inc.) with 15% SPS. In the morning of day 3 (at 70–72 h after insemination or ICSI), all cleaved embryos were group cultured in microdrops of a blastocyst medium (Sage BioPharma, Inc.) with 15% SPS. Expanding and expanded blastocysts underwent TE biopsy on day 5 or 6. The blastocyst quality was assessed immediately before TE biopsy, defined according to the criteria presented by Gardner and Schoolcraft (1999) [19]; only the blastocysts considered to be of a desirable quality (4, 5, 6, AB, BA, and BB) were subjected to biopsy. After TE biopsy, blastocysts were cultured in microdrops of a blastocyst medium with 15% SPS and a three-gas incubator until vitrification. The remaining average or poor quality blastocysts without TE biopsy were simultaneously vitrified.

### Trophectoderm biopsy

TE biopsy was performed on day 5 or 6 expanded blastocysts. The blastocysts for TE biopsy were loaded in culture dishes, which contained two to three microdroplets of a blastocyst medium (Sage BioPharma, Inc.) overlaid with paraffin oil (Vitrolife, Kungsbacka, Sweden). The blastocysts were held using a holding pipette (Humagen, Charlottesville, VA, USA), and laser pulses were used to punch a small hole in the ZP away from the inner cell mass to accommodate the passage of several TE cells. Approximately 5–10 TE cells detached from the ZP were aspirated into the biopsy pipette with smooth suction. The aspirated cells were detached from the blastocysts with several laser pulses combined with smooth suction. The detached cells were aspirated into the TE biopsy pipette and released into the biopsy drop. The biopsied TE cells were immediately placed in RNAse—DNAse-free polymerase chain reaction tubes. The blastocyst morphology following TE biopsy was typically collapsed.

### Comparative genomic hybridization analysis

The biopsied samples were collected with 2.5 μL of phosphate-buffered saline and subsequently amplified using the SurePlex DNA Amplification System (Illumina, Inc., San Diego, CA, USA). The samples were maintained at 4°C after adding 2.5 μL of a cell extraction buffer. For the extraction and random fragmentation of genomic DNA, the samples were mixed with an extraction cocktail and subsequently incubated at 75°C for 10 min and at 95°C for 4 min. In the preamplification step, the samples were mixed with a preamplification cocktail and incubated for one cycle at 95°C for 2 min and 12 cycles at 95°C for 15 s, 15°C for 50 s, 25°C for 40 s, 35°C for 30 s, 65°C for 40 s, and 75°C for 40 s. In the final step of amplification, the samples were mixed with amplification cocktail and incubated for one cycle at 95°C for 2 min and 14 cycles at 95°C for 15 s, 65°C for 1 min, and 75°C for 1 min. The amplified products were analyzed through 1.5% agarose gel electrophoresis, and successfully amplified DNA had lengths in the range of 100–1000 bp. The samples were subsequently processed for 24sure V3 microarrays (Illumina, Inc.).

The purified DNA was labeled with Cy3 and Cy5 fluorophores, and 8 μL of amplified or reference DNA was combined with 5 μL of a primer solution. The combined product was incubated at 94°C for 5 min and at 4°C for 5 min. Furthermore, 12 μL of a Cy3 or Cy5 labeling master mix was added to the DNA—primer solution and subsequently incubated for 2–4 h at 37°C. The labeled samples and reference DNA were hybridized to a microarray chip on 24sure V3 arrays at 47°C for 6–16 h. After hybridization, the slides were washed under the following conditions: gentle agitation (once) in a 2× saline—sodium citrate (SSC)/0.05% Tween20 buffer at room temperature for 10 min; once in a 1× SSC buffer at room temperature for 10 min; and once in a 0.1× SSC buffer at 60°C for 5 min, with a subsequent wash in a 0.1× SSC buffer at room temperature for 1 min. The washed slides were scanned after centrifugation at 170 x *g* for 3 min. The fluorescence intensity was detected using a laser scanner (InnoScan 710, Innopsys, Carbonne, France). BlueFuseMulti software was used for data processing. Arrays with a proportion of included clones > 85% and a signal-to-background ratio > 3 were considered and analyzed when the derivative log ratio was < 0.2. The blastocysts were first categorized as euploid or aneuploid. According to the 24sure V3 product description, the effective resolution of all 24 chromosomes for 24sure V3 is 10 Mb.

### Re-expansion grading at the time of vitrification

Many patients underwent TE biopsy (minimal, one and maximal, six) on the same day; therefore, the intervals between TE biopsy and vitrification were different among patients. All intervals between TE biopsy and vitrification were clearly recorded at the time of vitrification. The minimal and maximal intervals between TE biopsy and vitrification were 0.5 h and 6 h, respectively. The morphology of the biopsied blastocysts was immediately assessed at the time of vitrification, with the morphology defined according to the re-expansion grade. Briefly, biopsied blastocysts were divided into three groups on the basis of their degree of expansion status relative to the original blastocoel as follows: (1) a collapsed blastocyst without a significantly blastocoel (CB); (2) an expanding blastocyst with a blastocoel that is at least 3/4 of the volume of the embryo (RE); (3) a fully expanded blastocyst with a blastocoel completely filling the embryo (FE). Subsequently, the biopsied blastocysts were subjected to vitrification by using the Cryotech vitrification method (Repro-Support Medical Research Centre, Co. Ltd., 2-5-5-8F Shinjuku, Tokyo, Japan). Vitrification with Cryotech (Cryotech, Japan) was performed according to the protocol described by Gutnisky et al. (2013) [20].

### Warming protocol and frozen embryo transfer

The warming protocol with Cryotech described by Gutnisky et al. (2013) [20] was employed. One to two euploid blastocysts were selected for transfer, and warmed embryos were cultured in a blastocyst medium at 37°C (6% CO_2_ and 5% O_2_) for 1–2 h before the transfer. Embryo transfer procedures were performed as previously described [18]. The warmed blastocyst survival was checked at the timing of embryo transfer with either > 80% of cells intact or full re-expansion.

All patients underwent an artificial cycle for endometrial preparation on day 3 of their natural menstrual cycle and received transdermal estrogen patches. Each woman was administered the same regimen during the menstrual cycle: oral estradiol valerate (Estrade, Synmosa, Taipei, Taiwan) at 4 mg daily on days 3 and 4, 8 mg daily from days 5 to 7, 24 mg daily from days 8 to 13, 12 mg on day 14, and 8 mg daily from day 15 to the day of the pregnancy test. The transfer was performed on day 18 and required an endometrial thickness of at least 8 mm. If the endometrial thickness was < 8 mm, the transfer was canceled and shifted to the next cycle. On day 12 of the menstrual cycle, a progesterone injection (50 mg/day, Tai Yu Chemical & Pharmaceutical Co., Ltd, Taiwan) for 17 days was started for luteal phase support. Crinone (1.125 g, 8% gel; vaginal suppositories, Merck Serono, UK) application daily and oral dydrogesterone (10 mg; Duphaston, Abbott Biologicals B.V., the Netherlands) three times a day for 17 days, starting on day 13 of the menstrual cycle, were initiated. Estradiol and progesterone supplementation were continued for the following 2 weeks until the pregnancy test and, if the test result was positive, for another 4–5 weeks.

### Statistical analysis

All groups of individuals were assigned to study conditions and the analyses of embryo implantation and pregnancy rates were performed at the group level. Clinical pregnancies were diagnosed according to the presence of a gestational sac on transvaginal ultrasound 5 weeks after oocyte retrieval. A miscarriage between weeks 7 and 20 was defined as an abortion. The ongoing implantation rate was defined as the number of fetuses with heart activity after 20 weeks of gestation per transferred embryo. Differences between groups regarding age, the number of transferred embryos, and the number of viable embryos were analyzed using the Student *t* test. A chi-squared or Mann—Whitney test was used for comparing the clinical pregnancy, ongoing pregnancy, and implantation rates among groups. A difference of < 0.05 was considered significant. All calculations were performed using SPSS 17.0 (StatSoft Inc., Tulsa, USA).

## Results

### Expansion status of biopsied blastocysts during the culture interval between TE biopsy and vitrification

**Fig 2:**
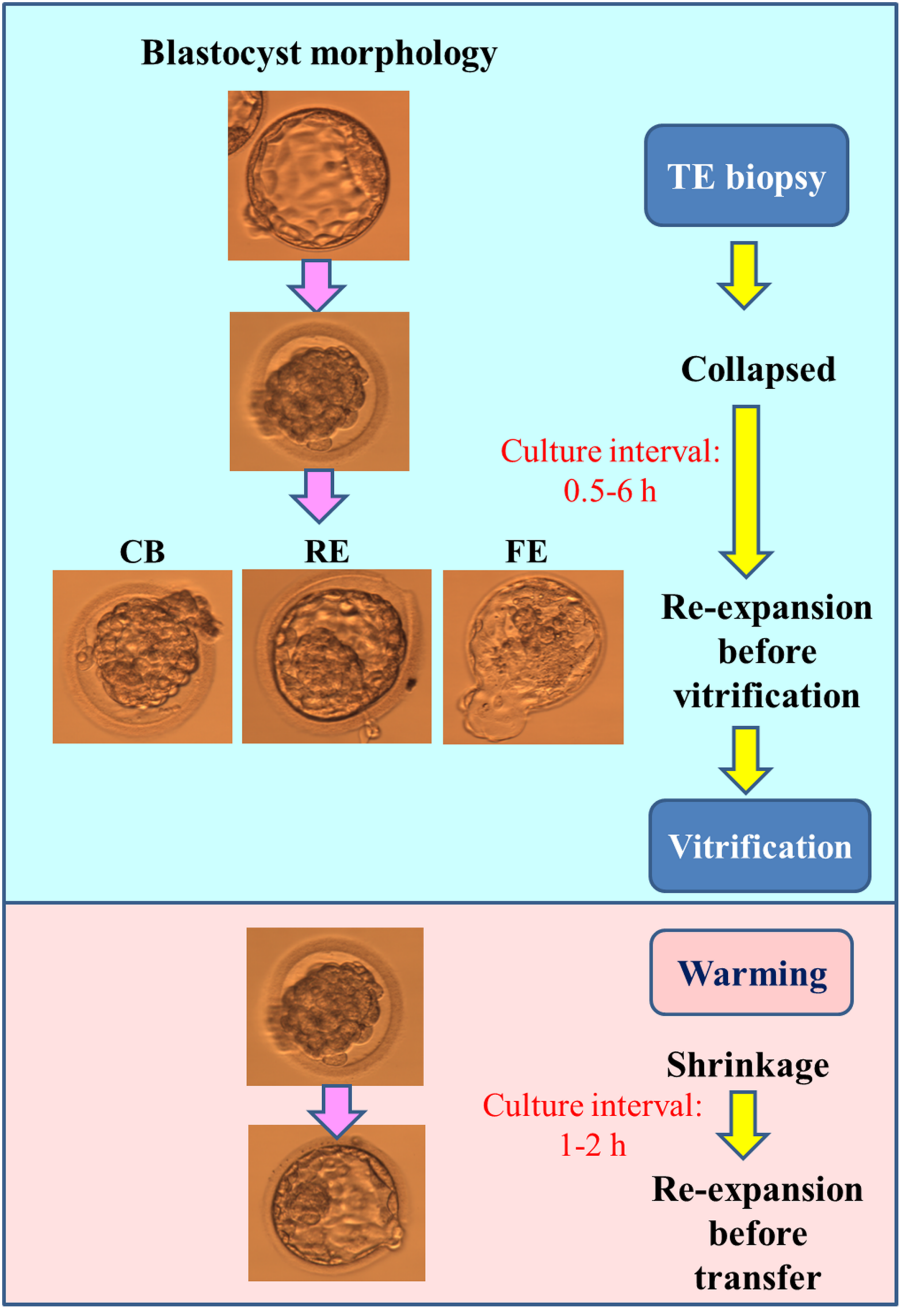
The morphological changes of blastocysts in the PGS-FET cycle. Abbreviation: CB, collapsed blastocyst; RE, re-expansion but not full expansion; FE, full re-expansion or hatching.

**Fig 3:**
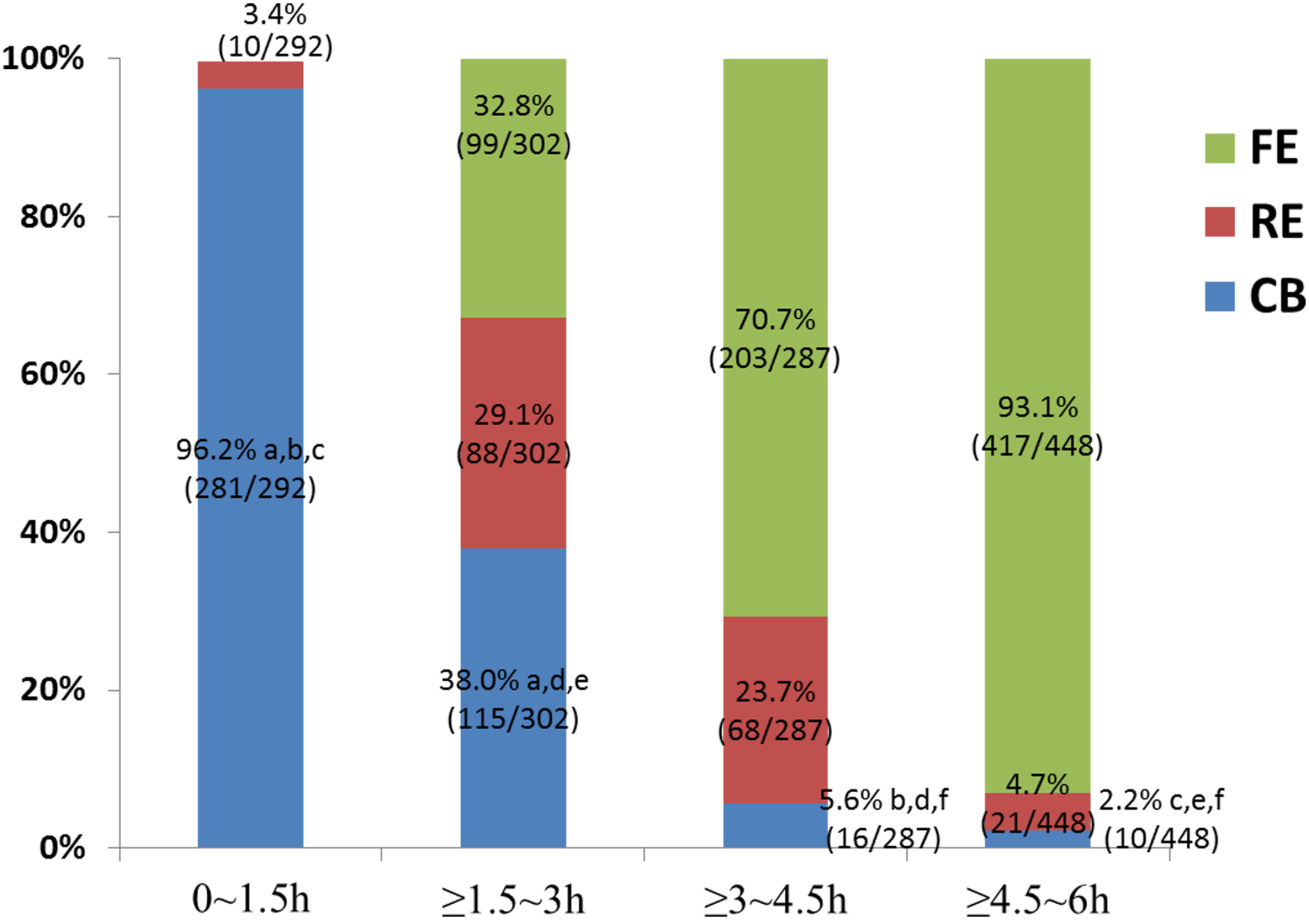
The expansion status of biopsied blastocysts cultivated *in vitro* for 0.5 to 6 h prior to cryopreservation. Abbreviation: CB, collapsed blastocyst; RE, re-expansion but not full expansion; FE, full re-expansion or hatching. ^a, b, c, d, e, f^Same superscript in the figure indicates statistical significance, p < 0.05.

There were 356 patients who underwent IVF-PGS protocols during the whole period of this study and a total of 223 patients were recruited with data for analysis (Fig 1). This study analyzed 1329 biopsied blastocysts from the 223 patients who underwent PGS (Table 1). The average patient age was 32.6 ± 3.0 years (range, 22–39 years). No blastocyst degeneration was observed after TE biopsy. The euploidy rate of the biopsied blastocysts was 43.3% (575/1329, Table 1). A total of 357 euploid blastocysts were warmed and transferred to the patients. All thawed blastocysts survived (100%, 357/357) and re-expanded at 1–2 h after warming. The implantation and clinical pregnancy rates in PGS-FET cycles were 60.2% (215/357) and 70.0% (156/223), respectively. The expansion status of the biopsied blastocysts at the time of vitrification is shown in Figs 2 and 3. None of the blastocysts re-expanded within 1 h after TE biopsy (data not showed). At 0–1.5 h after biopsy, 96.2% (281/292) of the blastocysts remained collapsed and showed a CB morphology, and at ≥ 1.5–3 h after biopsy, 38.0% (115/302) of the biopsied blastocysts showed a CB morphology. At ≥ 3–4.5 h after biopsy, only 5.6% (16/287) of the biopsied blastocysts showed a CB morphology. The CB percentages were significantly lower at ≥ 3–4.5 h (5.6%, 16/287) and ≥ 4.5–6 h (2.2%, 10/448) after biopsy than at 0–1.5 h (P < 0.05) and ≥ 1.5–3 h (P < 0.05) after biopsy. Moreover, ≥ 3 h after biopsy, up to 96.5% (709/735) of the biopsied blastocysts showed RE or FE; this percentage was significantly higher than those of the other groups (P < 0.001).

**Table 1:** Characteristics and clinical outcomes of patients undergoing PGS-FET.

Items	Results
Cycles (n)	223
Female age	32.6 ± 3.0 (22; 39)
Duration (year)	3.0 ± 3.2 (0; 15)
Oocyte retrieval no.	17.6 ± 4.7 (9; 27)
Blastocyst no.	8.5 ± 2.2 (4; 13)
Biopsied and vitrified blastocyst no.	5.9 ± 1.6 (3; 9)
Euploidy rate of biopsied blastocyst	43.3% (575/1329)
Warmed blastocyst no.	357
Survival rate	100% (357/357)
Warmed and transferred blastocyst no.	1.6 ± 0.5 (1; 2)
Implantation rate	60.2% (215/357)
Pregnancy rate	70.0% (156/223)
Ongoing pregnancy rate	63.7% (142/223)
Abortion rate	8.9% (14/157)

In the table, percentages are used to describe categorical data and mean ± SD (min; max) used to describe a set of continuous data.

### Implantation potential of biopsied blastocysts with different culture intervals between TE biopsy and vitrification

According to the culture intervals of the transferred blastocysts between TE biopsy and vitrification, the PGS-FET cycles were divided into two groups: < 3 h culture interval (n = 44 cycles) and ≥ 3 h culture interval (n = 179 cycles; Table 2) groups. The implantation (63.1%, 183/290), clinical pregnancy (73.7%, 132/179), and ongoing pregnancy rates (67.0%, 120/179) in the ≥ 3 h culture interval group were significantly higher than those in the < 3 h culture interval group (47.8%, 32/67; 54.5%, 24/44; and 50.0%, 22/44 and P = 0.021, 0.013, and 0.035, respectively; Table 2).

**Table 2:** Clinical outcomes after embryo transfer of euploid blastocysts with different culture intervals before vitrification.

	< 3h	≥ 3h	P value
Cycles (n)	44	179	-
Female age	32.6 ± 4.0	32.6 ± 4.3	0.859
ET no.	1.5 ± 0.5	1.6 ± 0.5	0.303
IR (%)	47.8 (32/67)	63.1 (183/290)	0.021
Clinical PR (%)	54.5 (24/44)	73.7 (132/179)	0.013
Ongoing PR (%)	50.0 (22/44)	67.0 (120/179)	0.035
Abortion rate (%)	8.3 (2/24)	9.2 (12/131)	0.134

In the table, percentages are used to describe categorical data and mean ± SD used to describe a set of continuous data.

Abbreviation: ET, embryo transfer; PR, pregnancy rate; IR, implantation rate.

### Simultaneous effects of culture intervals and the embryo expansion status after TE biopsy on the implantation potential of biopsied blastocysts

By combining two factors, namely the culture intervals and expansion status at the time of vitrification, to assess their effects on clinical outcomes, the patients were subdivided into four groups: transferred blastocysts cultured for < 3 h before vitrification and still showing a CB morphology (Group I, n = 34 cycles); transferred blastocysts cultured for < 3 h before vitrification and showing a RE or FE morphology (Group II, n = 10 cycles); transferred blastocysts cultured for ≥ 3 h before vitrification and showing a CB morphology (Group III, n = 6 cycles); and transferred blastocysts cultured for ≥ 3 h before vitrification and showing a RE or FE morphology (Group IV, n = 173 cycles; Table 3). The implantation, clinical, and ongoing pregnancy rates in Group IV (63.7%, 179/281; 74.0%, 128/173; and 67.1%, 116/173, respectively) were significantly higher than those in Group I (45.3%, 24/53; 50.0%, 17/34; and 41.2%, 14/34, respectively. P = 0.012, 0.005, and 0.004, respectively). The ongoing pregnancy rates in Groups II (70.0%, 7/10) and III (66.7%, 4/6) were higher, than those in Group I, but the difference was nonsignificant (P = 0.109 and 0.247, respectively; Table 3).

**Table 3:** Clinical outcomes after embryo transfer of enuploid blastocysts simultaneously considering different re-expansion status and culture intervals before vitrification.

Intervals	< 3h		≥ 3h	
Morphology	Group I (CB)	Group II (RE or FE)	Group III (CB)	Group IV (R or FE)
Cycles (n)	34	10	6	173
Female age (mean±SD)	33.1 ± 4.1	31.2 ± 3.2	30.0 ± 2.4	32.7 ± 4.4
ET no.	1.6 ± 0.5	1.4 ± 0.5	1.5 ± 0.5	1.6 ± 0.5
IR (%)	45.3 ^a^ (24/53)	57.1 (8/14)	44.4 (4/9)	63.7^a^ (179/281)
Clinical PR (%)	50.0 ^b^ (17/34)	70.0 (7/10)	66.7 (4/6)	74.0 ^b^ (128/173)
Ongoing PR (%)	41.2 ^c^ (14/34)	70.0 (7/10)	66.7 (4/6)	67.1 ^c^ (116/173)
AR (%)	11.8 (2/17)	0 (0/7)	0 (0/4)	9.4 (12/128)

In the table, percentages are used to describe categorical data and mean ± SD used to describe a set of continuous data.

Abbreviation: ET, embryo transfer; PR, pregnancy rate; IR, implantation rate; AR, abortion rate.

^a^Same superscript in the same row indicates statistical significance, p < 0.05,

^b, c^ Same superscript in the same row indicates statistical significance, p < 0.01.

## Discussion

When an expanded blastocyst is subjected to the PGS—FET protocol, shrinkage majorly occurs at two stages (Fig 2), namely after TE biopsy and after warming. The re-expansion grade after warming has been used to indicate blastocyst survivability and quality after vitrification [21–26] and the clinical pregnancy rate of rapidly re-expanding blastocysts (within 2–4 h) was more than double the rate of slowly re-expanding blastocysts [21]. In this study, blastocyst degeneration was not observed after TE biopsy and only euploid blastocysts were selected for embryo transfer. All of biopsied blastocysts appeared to survive and re-expand 1–2 h after warming (Table 1). According to previous studies [21, 27], the biopsied blastocysts in this study were expected to yield similar implantation outcomes among groups. However, the biopsied blastocysts in the RE or FE groups showed significantly higher implantation and pregnancy rates than did those in the CB group (data not showed). Moreover, TE biopsy, embryo vitrification and warming, and embryo transfer were performed according to the same standard protocol. All blastocyst vitrification protocols or TE biopsy in this study were performed by the same embryologists to reduce operator impact. Therefore, this study focused on the influence of the vitrification timing and expansion status of the biopsied blastocysts.

A significant collapse occurred as a result of laser assisted hatching on day 5 or day 6 to biopsy the blastocyst and the re-expansion occurred after culturing. This study indicated that the re-expansion grades of the biopsied blastocysts increased following an extended culture period between TE biopsy and vitrification (Fig 3). None of the blastocysts re-expanded within 1 h after TE biopsy; however, more than 96.5% of the blastocysts reached a RE or FE status after ≥ 3 h of culture. Moreover, the post-FET implantation and pregnancy rates were significantly higher in the ≥ 3 h culture interval group (Table 2), indicating that the culture interval before vitrification affected the post-FET clinical outcomes.

Technically, the removal of multiple cells from the TE of a blastocyst results in a decrease in the cell number and embryo integrity that may hinder subsequent embryo development. Ugajin *et al*. (2010) [28] reported adverse effects of blastomere removal on embryonic development, and volume reduction seems to prolong hatching in the blastomere removal group. However, blastocyst expansion during 3–6 h of cultivation after biopsy was not associated with increased cell number derived from cell divisions. Hardy *et al*. (1989) [29] concluded that the average doubling time of human embryos at the blastocyst stage on days 5, 6, and 7 is 24 h. Thus, the improvement in clinical outcomes achieved using a prolonged culture period after biopsy may not be associated with an increased cell number. Actually, TE biopsy appears to cause the rupture of the TJs between TE cells, which are responsible for paracellular sealing for retaining Na^+^ and water molecules inside the blastocoel [15]. The TJs between TE cells play a crucial role in intercellular integrity, embryo amino acid turnover [15, 30], embryonic growth [31] and implantation [32]. The paracellular sealing and sodium pump (Na+/K+-ATPase) facilitates gradually increasing fluid accumulation in the blastocoel [16], resulting in increased pressure on both the TE and ZP to increase blastocoel size to form an expanded blastocyst. The re-expansion of the biopsied blastocysts may be associated with fluid re-accumulation in the blastocoel before vitrification, and blastocysts with a ≥ 3 h culture interval prior to vitrification exhibited a sufficient re-expansion rate and more favorable clinical outcomes.

The parallel advances in blastocyst culture and introduction of vitrification in the routine management of IVF cycles enabled TE biopsy to be performed with high efficiency and minimal risks [3, 12]; however, for blastocyst vitrification, an ice-free glassy state is achieved using high concentrations of cryoprotectants and rapidly cooling the embryos to liquid nitrogen temperature. A major difficulty with this approach is that the high cryoprotectant concentrations required for avoiding crystal formation increase the risk of osmotic and toxic damage [33]. Although vitrification shows a high survival rate, the reduction of osmotic shock and toxicity by employing an appropriate equilibration step or cryoprotectant exposure time is critical to improving survival rates and embryo development [34–37]. Moreover, Gajda and Smorag (2002) [38] reported that the vitrification solution was more toxic to hatched porcine blastocysts (a broken zona) than to expanded blastocysts. Thus, investigating an optimal recovery status of biopsied blastocysts with broken zona pellucida and TJ-impaired TE cells would be critical for equilibration with cryoprotectants. According to the elevated clinical outcomes in this study, we suggested that culturing for ≥ 3 h might alleviate TJ impairment in the biopsied blastocysts and further enhance the equilibration efficiency during vitrification.

In this study, some biopsied blastocysts (n = 10) that reached RE or FE before 3 h of culture (Group II; Table 3) had acceptable efficiencies in implantation, clinical pregnancy, and ongoing pregnancy, suggesting that the rapid re-expansion of the biopsied blastocysts was associated with the higher repair rate of paracellular sealing. By contrast, some biopsied blastocysts (n = 6) still showed a CB morphology after 3 h of culture (Group III; Table 3). Group III showed a low implantation rate (44.4%), similar to the CB blastocysts with a <3 h culture interval (45.3%; Group I). Therefore, we assumed that the biopsied blastocysts unable to recover from TE biopsy after a prolonged culture period were associated with more damage to paracellular sealing or a lower rate of repair. However, the small sample sizes of Groups II and III implied lower statistical power than that for Groups I and IV.

In conclusion, this study evaluated the effects of the culture intervals between TE biopsy and vitrification and expansion status of biopsied blastocysts at the time of vitrification on clinical outcomes after PGS—FET. The results reveal that the biopsied blastocysts required a minimal culture period of 3 h before vitrification, and selection of RE or FE biopsied blastocysts for embryo transfer is beneficial to clinical outcomes. These results are critical for achieving higher implantation rates in PGS—FET protocols than in conventional protocols without an additional culture period after biopsy.

## Supporting information

S1 File Original minimal data. (SAV)

S2 File Trend checklist. (PDF)

S3 File Original—CS14124-013-study protocol. (DOC)

S4 File Clinical trail protocol-translation. (DOC)
