# Supplementary material for: Optimal timing of blastocyst vitrification after trophectoderm biopsy for preimplantation genetic screening
Source: PLoS One. 2017 Oct 5;12(10):e0185747. doi: 10.1371/journal.pone.0185747 (PMC5628850; doi:10.1371/journal.pone.0185747)
Supplement: S3 File — (DOC) [file pone.0185747.s003.doc]

計畫書版本︰1.0

生效日期︰103-11-10

| 本院案號 | CS 14124 |
| --- | --- |
| 計畫名稱 | 中文: 不同胚胎發育時期進行胚胎植入前遺傳診斷及篩檢之臨床結果 |
|  | 英文: Retrospective study on clinical results of preimplantation genetic diagnosis and screening at different embryo developmental stage |
| 主持人 | 李茂盛 (計畫主持人請簽署，表示了解下列撰寫說明。) |
| 研究機構 | 中山醫學大學 醫學研究所 |

- 1. 研究背景及重要性

胚胎植入前遺傳診斷(preimplantational genetic diagnosis)及胚胎植入前遺傳篩檢 (preimplantational genetic screen ) 是針對有遺傳疾病家族史之病患、夫婦本身具有遺傳疾病或可能產生染色體異常的下一代，所進行的胚胎診斷技術。欲進行胚胎植入前遺傳診斷必須需先進行人工生殖療程，其過程包括誘導排卵、取卵、體外受精及胚胎培養，至胚胎成長到六至八細胞或培養成囊胚期的階段，在顯微鏡下進行胚胎切片，取一或數個細胞，將切片細胞進行遺傳診斷，選擇無遺傳疾病之胚胎植入子宮腔，以避免生下有遺傳疾病的後代。

胚胎植入前遺傳診斷可以減少懷孕時染色體異常的胎兒著床後，又要面對流產的痛苦，並且可以避免進行試管嬰兒療程的夫婦植入或生下遺傳異常的下一代，藉此大幅降低生下具有遺傳疾病後代的機率，是推廣優生學及降低社會成本很重要的檢測技術，但是國內尚無大量的臨床統計數據以資參考此技術及臨床結果對國人的影響。

- 1. 研究目的

進行分裂期胚胎檢測或囊胚期胚胎檢測及染色體異常是進行植入前遺傳診斷或篩檢的成效以做為臨床的參考的重要依據，但國內研究尚無完整的分析。本計畫將比較分析不同胚胎發育階段進行胚胎檢測之基因或染色體異常及臨床結果。

- 1. 研究對象及實施方法

1. 研究設計

本研究為回溯性研究，調閱自民國90年起迄今，曾於李茂盛婦產科進行試管嬰兒療程合併胚胎植入前遺傳診斷或篩選之受術夫婦病例資料，摘錄其夫婦之年齡、不孕或求診原因、卵子及胚胎品質、胚胎遺傳診斷或遺傳篩選之結果、懷孕率等資料，以英文字母及數字重新編碼，去連結。再統計這些資料的關連性。

1. 預計執行研究期間及預計進度︰

研究期間: 103年12月 1日 至 104年11月30日

預計進度:

調閱資料收集整理: 103年12月 至 104年5月

統計分析: 104年 6月 至 104年 8月

結果討論: 104年 9月 至 104年 10月

撰寫結案報告: 104年 10月 至 104年11月

1. 納入條件 (inclusion criteria)

自民國90年起曾在李茂盛婦產科進行試管嬰兒療程合併胚胎植入前遺傳診斷或篩選之受術夫婦

1. 排除條件 (exclusion criteria)

具有重大疾病之受術夫婦

1. 中途退出條件 (withdrawal criteria)

無

四、指標（療效）評估方式

1. 主要（療效）指標

分析胚胎品質及懷孕結果與胚胎遺傳診斷或篩檢的相關性

1. 次要（療效）指標

無

1. 其它

無

五、研究對象人數（請說明計算方式）及資料統計處理方式

研究對象為曾在李茂盛婦產科診所進行試管嬰兒合併胚胎遺傳診斷或 篩檢療程的受術夫婦，人數: 500 對受術夫婦

六、研究對象招募方式︰

療程已結束，病患回診率不高，故採回溯性研究，直接篩選病歷資料去連結後，進行統計。

七、預期成果及主要效益

分析進行植入前遺傳診斷或篩檢的胚胎品質、懷孕率與胚胎植入前遺傳診斷的結果對照，以做為臨床醫療的參考。

八、研發成果之歸屬及運用

視研究成果於相關醫學會或期刊發表，供學術及臨床應用參考，對主持人、研究機構與研究對象等無經濟利益。

九、研究對象可能的副作用、與之後續追蹤處理方式及必要之復健計畫。

本計畫僅摘錄植入前遺傳診斷或篩檢的胚胎品質、懷孕率與胚胎植入前遺傳診斷的結果統計，不會對研究對象產生醫療上的副作用亦不適用後續追蹤處理方式及復健計畫。

十、研究對象權益的保障及可能引起之損害及其救濟措施。

本計畫僅摘錄植入前遺傳診斷或篩檢的胚胎品質、懷孕率與胚胎植入前遺傳診斷的結果統計，研究對象有權可在任何時間拒絕、退出試驗，且不會遭到處罰或損及權益。

**十一、監測與稽核試驗進行之規劃 (Data & Safety Monitoring Plan)︰**

本計畫為低風險又設計單純的研究計畫，單純查閱病歷回溯統計臨床結果，不會影響研究對象身心安全，無預期不良事件與反應，確保資料正確及計畫順利執行，於摘錄須統計的資料後，數字及英文字母取代受試者去連結，依預定執行進度進行試驗。

十二、研究人力及相關設備需求

研究人力為主持人須電腦統計軟體

十三、參與試驗之人員（列表），可在檢附試驗主持人及主要協同人員之學、經歷及其所受訓練之背景資料、研究成果及發表論文。

| 序號 | 參與者姓名 | 職稱 | 預計擔任工作 |
| --- | --- | --- | --- |
| 1 | 李茂盛 | 教授 | 統籌及撰寫計畫 |
| 2 | 鄭恩惠 | 研究員 | 調閱病歷、統計資料 |

十四、預計可能遭遇的困難與解決途徑

如病例資料遺失或記錄不完整可能導致無法蒐集足夠之資料分析時，解決途徑則僅選擇完整資料者統計。

十五、研究人員利益衝突之揭露︰

研究經費來源為主持人自行付擔，我們沒有利益衝突。

十六、試驗藥品、醫療器材或醫療技術說明︰

不適用

十七參考資料如以下文獻：

# (1). [Rubio C](http://www.ncbi.nlm.nih.gov/pubmed?term=Rubio C%5BAuthor%5D&cauthor=true&cauthor_uid=23394777), [Rodrigo L](http://www.ncbi.nlm.nih.gov/pubmed?term=Rodrigo L%5BAuthor%5D&cauthor=true&cauthor_uid=23394777), [Mir P](http://www.ncbi.nlm.nih.gov/pubmed?term=Mir P%5BAuthor%5D&cauthor=true&cauthor_uid=23394777), [Mateu E](http://www.ncbi.nlm.nih.gov/pubmed?term=Mateu E%5BAuthor%5D&cauthor=true&cauthor_uid=23394777), [Peinado V](http://www.ncbi.nlm.nih.gov/pubmed?term=Peinado V%5BAuthor%5D&cauthor=true&cauthor_uid=23394777), [Milán M](http://www.ncbi.nlm.nih.gov/pubmed?term=Milán M%5BAuthor%5D&cauthor=true&cauthor_uid=23394777), [Al-Asmar N](http://www.ncbi.nlm.nih.gov/pubmed?term=Al-Asmar N%5BAuthor%5D&cauthor=true&cauthor_uid=23394777), [Campos-Galindo I](http://www.ncbi.nlm.nih.gov/pubmed?term=Campos-Galindo I%5BAuthor%5D&cauthor=true&cauthor_uid=23394777), [Garcia S](http://www.ncbi.nlm.nih.gov/pubmed?term=Garcia S%5BAuthor%5D&cauthor=true&cauthor_uid=23394777), [Simón C](http://www.ncbi.nlm.nih.gov/pubmed?term=Simón C%5BAuthor%5D&cauthor=true&cauthor_uid=23394777). [Fertil Steril.](http://www.ncbi.nlm.nih.gov/pubmed/23394777) Use of array comparative genomic hybridization (array-CGH) for embryo assessment: clinical results. 2013; (13)00137-4

# (2). [**Harper JC**](http://www.ncbi.nlm.nih.gov/pubmed?term=Harper JC%5BAuthor%5D&cauthor=true&cauthor_uid=21748341), [**Sengupta SB**](http://www.ncbi.nlm.nih.gov/pubmed?term=Sengupta SB%5BAuthor%5D&cauthor=true&cauthor_uid=21748341). **Preimplantation** **genetic** diagnosis: state of the art 2011. [**Hum Genet.**](http://www.ncbi.nlm.nih.gov/pubmed/21748341) 2012; 131(2):175-86.

(3). [Capalbo A](http://www.ncbi.nlm.nih.gov/pubmed?term=Capalbo A%5BAuthor%5D&cauthor=true&cauthor_uid=23148203), [Bono S](http://www.ncbi.nlm.nih.gov/pubmed?term=Bono S%5BAuthor%5D&cauthor=true&cauthor_uid=23148203), [Spizzichino L](http://www.ncbi.nlm.nih.gov/pubmed?term=Spizzichino L%5BAuthor%5D&cauthor=true&cauthor_uid=23148203), [Biricik A](http://www.ncbi.nlm.nih.gov/pubmed?term=Biricik A%5BAuthor%5D&cauthor=true&cauthor_uid=23148203), [Baldi M](http://www.ncbi.nlm.nih.gov/pubmed?term=Baldi M%5BAuthor%5D&cauthor=true&cauthor_uid=23148203), [Colamaria S](http://www.ncbi.nlm.nih.gov/pubmed?term=Colamaria S%5BAuthor%5D&cauthor=true&cauthor_uid=23148203), [Ubaldi FM](http://www.ncbi.nlm.nih.gov/pubmed?term=Ubaldi FM%5BAuthor%5D&cauthor=true&cauthor_uid=23148203), [Rienzi L](http://www.ncbi.nlm.nih.gov/pubmed?term=Rienzi L%5BAuthor%5D&cauthor=true&cauthor_uid=23148203), [Fiorentino F](http://www.ncbi.nlm.nih.gov/pubmed?term=Fiorentino F%5BAuthor%5D&cauthor=true&cauthor_uid=23148203). Sequential comprehensive chromosome analysis on polar bodies, blastomeres and trophoblast: insights into female meiotic errors and chromosomal segregation in the preimplantation window of embryo development. [Hum Reprod.](http://www.ncbi.nlm.nih.gov/pubmed/23148203) 2013 Feb;28(2):509-18.

十八、研究經費需求及經費來源揭露，及經費預算表

此為學術研究案，所有經費由主持人自行負擔

| 預算明細表 | | | |
| --- | --- | --- | --- |
| ※試驗開始前，醫院行政費用需一次結清 (IRB送審費用另計) | | | |
| 試驗項目 | 摘要 | 金額 | 說明(請註明計算方式) |
| 人事費用 | 試驗主持人費 | 0元 | 現有人力 |
| 人事費用 | 研究護士原有人力/助理 | 0元 | 現有人力 |
| 檢驗費用 | 試劑耗材、檢驗費 | 0元 | N/D: 回溯性研究 |
| 受試者費用 | 掛號費、診察費等 | 0元 | N/D: 回溯性研究 |
| 受試者費用 | 車馬費 | 0元 | N/D: 回溯性研究 |
| 雜支 | 郵電、紙張、影印費用等 | 2500元 | A4紙張200元、影印、文具及電腦周邊耗材 2300元 |
| 其它 | IRB審查費 | 2000元 | 院內學術案 |
| 藥品管理費 | 藥品及醫療器材管理費用 | 0元 | **無試驗藥品** |
| 試驗經費(A) | 以上項目加總 | 4500元 |  |
|  | | | |
| 行政管理費(B) | 醫院行政管理費用  為總經費之10%:  **(B)=〔(A)／0.9〕－(A)** | 500元 | **計算方式說明：〔試驗經費(A) ／0.9〕－試驗經費(A)，計算結果請四捨五入至個位整數** |
|  | | | |
| 總經費(C) | (C)＝(A)+(B) | 5000元 | 總經費(C)=試驗經費(A)+行政管理費(B) |
