# Supplementary material for: Optimal timing of blastocyst vitrification after trophectoderm biopsy for preimplantation genetic screening
Source: PLoS One. 2017 Oct 5;12(10):e0185747. doi: 10.1371/journal.pone.0185747 (PMC5628850; doi:10.1371/journal.pone.0185747)
Supplement: S4 File — (DOC) [file pone.0185747.s004.doc]

Project version︰1.0

Effective date︰2014-Nov.-10

| Study ID Numbers | CS 14124 |
| --- | --- |
| Title | Retrospective study on clinical results of preimplantation genetic diagnosis and screening at different embryo developmental stage |
| Study Chair | Maw-Sheng Lee, PhD |
| Official Title | Institute of Medicine, Chung Shan Medical University |

- 1. The research background and importance

Preimplantational genetic diagnosis (PGD) and preimplantational genetic screen (PGS) is a diagnosis technique for patients to avoid transferred embryos with genetic diseases or chromosomal abnormalities. Before embryo selection by PGS diagnosis, embryo must be carried out artificial reproductive treatment protocols, the process including: ovarian stimulation, oocyte pick up, in vitro fertilization and embryo culture. When embryos grow to six to eight cells or cultured into the stage of blastocyst, take one or several cells from the biopsy of blastomeres for genetic diagnosis. After PGS, selecting the embryo without genetic disease to transfer can avoid the birth of genetic disease.

Preimplantation genetic diagnosis can reduce the risk of abortion caused by the implantation of chromosomal abnormalities, reduced the experience of abortion, avoid the risk of epigenetic influence of in vitro fertilization and even further reduce the social cost. However, there is not a lot of clinical data associated with the influence of this technique and clinical results on people in Taiwan.

- 1. Purpose

The detection of chromosomal abnormalities on embryos or blastocysts is an important clinical diagnosis or screening before implantation. However, there is no complete analysis of domestic studies. This study will analyze the relationship between the genes or chromosomal abnormalities and clinical outcomes at different stages of embryo.

- 1. The research object and methods

1. Study design

This study is a retrospective study; data collection including all patients underwent IVF and PGS or PGD from 2001 till now in the Lee's Women Hospital. Data including infertility history, age, the history of the treatment, the quality of the oocyte and embryo, the PGD or PGS results, pregnancy rate and other information. All patients’ records will be digital re-encoding to the English alphabet to link. And then statistics of the relevance of these data will be analyzed.

1. Expected implementation of study period and expected progress︰

Study duration: from 2014 Dec. to 2015 Nov.

Expected progress:

Data collection: from 2014 Dec. to 2015 May.

Data analysis: from 2015 Jun. to 2015 Aug.

Discussion of results: from 2015 Sep. to 2015 Sep.

Writing the manuscript: from 2015 Oct. to 2015 Nov.

1. inclusion criteria

All patients underwent IVF and PGS or PGD from 2001 till now in the Lee's Women Hospital.

1. exclusion criteria

The couples who have a sever disease

1. withdrawal criteria

N/A

四、Index (efficacy) assessment method

1. primary indicator

Analysis of the relationship between embryo quality and pregnancy outcome and genetic diagnosis or screening of embryos.

1. secondary indicator

N/A

1. others

N/A

五、sample size and statistic method

The couples underwent IVF and PGS or PGD from 2001 till now in the Lee's Women Hospital.，sample size: 500 couples

六、Recruitment method︰

Patients' IVF treatment protocols have been completed and finished. Because most of the patient do not will return the clinic after completing of the treatment, it is necessary to take the retrospective study. After screening and recording all medical records of patients, the analysis results will undergo by statistical methods.

七、Expected results and main benefits

Analysis the relationship between preimplantation genetic diagnosis or screening of embryonic quality and pregnancy rate and according these results we want to find a reference of clinical diagnosis to select embryos for transfer after PGS.

八、Results attribution and use in clinic

The research results will be published in relevant medical societies or journals for academic and clinical application. This study has no economic interest for the project chair, the research organization and the research object。

九、The possible side effects of the study object, follow-up treatment and the necessary rehabilitation plan

This retrospective study only analysis the results of pre-implantation genetic diagnosis and screening, and does not have any medical side effects on the subject and does not apply to subsequent follow-up and rehabilitation plan.

十、The protection of the object rights and the possible harm and the relief measures

This retrospective study only analysis the results of pre-implant genetic diagnosis or screening. The subject is entitled to refuse, withdraw from the trial at any time and will not be penalized or damage to equity.

**十一、Data & Safety Monitoring Plan︰**

This program is designed for lowest risk for patients and it is a simple research program. It is simple to check the clinical results of medical records. It will not affect the physical and mental safety of the subjects. There is no expectation of adverse events and reactions to ensure the correctness of the data and the smooth implementation of the plan. The number of letters and English letters will replace the subjects’ results to reduce the risk of exposure of personal’s treatment history and the implementation test will be accord to the scheduled progress.

十二、The needs of researchers and related equipment

The major researcher is the planning chairman and need computer statistics software.

十三、Participants in the trial (list), in the inspection of the test host and the main synergy of the school, experience and training background information, research results and published papers.

|  | Participants | job title | jobs |
| --- | --- | --- | --- |
| 1 | Maw-Sheng Lee | professor | Co-ordinate and write a plan |
| 2 | En-Hui Cheng | researcher | Access to medical records, statistics |

十四、Difficulties and solutions that are expected to be encountered

If the medical records are lost or incomplete records may lead to the collection of sufficient data analysis, the solution is only selecting the complete data statistics.

十五、Explanations of the Conflicts of Interests of Researchers︰

Research funding sources are from the planning chairman, researchers have no conflict of interest。

十六、Test medicine, medical equipment or medical technology︰

Not applicable.

十七 Reference：

# (1). [Rubio C](http://www.ncbi.nlm.nih.gov/pubmed?term=Rubio C%5BAuthor%5D&cauthor=true&cauthor_uid=23394777), [Rodrigo L](http://www.ncbi.nlm.nih.gov/pubmed?term=Rodrigo L%5BAuthor%5D&cauthor=true&cauthor_uid=23394777), [Mir P](http://www.ncbi.nlm.nih.gov/pubmed?term=Mir P%5BAuthor%5D&cauthor=true&cauthor_uid=23394777), [Mateu E](http://www.ncbi.nlm.nih.gov/pubmed?term=Mateu E%5BAuthor%5D&cauthor=true&cauthor_uid=23394777), [Peinado V](http://www.ncbi.nlm.nih.gov/pubmed?term=Peinado V%5BAuthor%5D&cauthor=true&cauthor_uid=23394777), [Milán M](http://www.ncbi.nlm.nih.gov/pubmed?term=Milán M%5BAuthor%5D&cauthor=true&cauthor_uid=23394777), [Al-Asmar N](http://www.ncbi.nlm.nih.gov/pubmed?term=Al-Asmar N%5BAuthor%5D&cauthor=true&cauthor_uid=23394777), [Campos-Galindo I](http://www.ncbi.nlm.nih.gov/pubmed?term=Campos-Galindo I%5BAuthor%5D&cauthor=true&cauthor_uid=23394777), [Garcia S](http://www.ncbi.nlm.nih.gov/pubmed?term=Garcia S%5BAuthor%5D&cauthor=true&cauthor_uid=23394777), [Simón C](http://www.ncbi.nlm.nih.gov/pubmed?term=Simón C%5BAuthor%5D&cauthor=true&cauthor_uid=23394777). [Fertil Steril.](http://www.ncbi.nlm.nih.gov/pubmed/23394777) Use of array comparative genomic hybridization (array-CGH) for embryo assessment: clinical results. 2013; (13)00137-4

# (2). [**Harper JC**](http://www.ncbi.nlm.nih.gov/pubmed?term=Harper JC%5BAuthor%5D&cauthor=true&cauthor_uid=21748341), [**Sengupta SB**](http://www.ncbi.nlm.nih.gov/pubmed?term=Sengupta SB%5BAuthor%5D&cauthor=true&cauthor_uid=21748341). **Preimplantation** **genetic** diagnosis: state of the art 2011. [**Hum Genet.**](http://www.ncbi.nlm.nih.gov/pubmed/21748341) 2012; 131(2):175-86.

(3). [Capalbo A](http://www.ncbi.nlm.nih.gov/pubmed?term=Capalbo A%5BAuthor%5D&cauthor=true&cauthor_uid=23148203), [Bono S](http://www.ncbi.nlm.nih.gov/pubmed?term=Bono S%5BAuthor%5D&cauthor=true&cauthor_uid=23148203), [Spizzichino L](http://www.ncbi.nlm.nih.gov/pubmed?term=Spizzichino L%5BAuthor%5D&cauthor=true&cauthor_uid=23148203), [Biricik A](http://www.ncbi.nlm.nih.gov/pubmed?term=Biricik A%5BAuthor%5D&cauthor=true&cauthor_uid=23148203), [Baldi M](http://www.ncbi.nlm.nih.gov/pubmed?term=Baldi M%5BAuthor%5D&cauthor=true&cauthor_uid=23148203), [Colamaria S](http://www.ncbi.nlm.nih.gov/pubmed?term=Colamaria S%5BAuthor%5D&cauthor=true&cauthor_uid=23148203), [Ubaldi FM](http://www.ncbi.nlm.nih.gov/pubmed?term=Ubaldi FM%5BAuthor%5D&cauthor=true&cauthor_uid=23148203), [Rienzi L](http://www.ncbi.nlm.nih.gov/pubmed?term=Rienzi L%5BAuthor%5D&cauthor=true&cauthor_uid=23148203), [Fiorentino F](http://www.ncbi.nlm.nih.gov/pubmed?term=Fiorentino F%5BAuthor%5D&cauthor=true&cauthor_uid=23148203). Sequential comprehensive chromosome analysis on polar bodies, blastomeres and trophoblast: insights into female meiotic errors and chromosomal segregation in the preimplantation window of embryo development. [Hum Reprod.](http://www.ncbi.nlm.nih.gov/pubmed/23148203) 2013 Feb;28(2):509-18.

十八、Research funding needs and funding sources to disclose, and the budget table

This is an academic research case, all the funds borne by the planning chairman

| Budget schedule | | | |
| --- | --- | --- | --- |
| ※Before the start of the trial, the hospital administrative expenses need to finish to paid. (IRB examination fee separately) | | | |
| Items | abstract | Fee (NT dollars) | explanation |
| Researcher cost | Chairman cost | 0 |  |
| Researcher cost | Researcher cost | 0 |  |
| Medical examination fee | Reagent, inspection fees | 0 | N/D: retrospective study |
| Subject cost | Registration fee | 0 | N/D: retrospective study |
| Subject cost | Traveling expenses | 0 | N/D: retrospective study |
| Others | Post and telecommunications, paper, photocopying costs | 2500 NT | A4紙 |
| Others | IRB examination fee | 2000 NT |  |
| Medical management | Drug and medical equipment management fees | 0 | Not applicable |
| Trail funding fee (A) | total | 4500 NT |  |
|  | | | |
| Management fee (B) | **(B)=〔(A)／0.9〕－(A)** | 500 NT |  |
|  | | | |
| Total fee (C) | (C)＝(A)+(B) | 5000 NT |  |
